# Bilateral Phacomatosis Pigmentovascularis in a Young Male with Developmental Glaucoma and Varicose Veins

**DOI:** 10.5005/jp-journals-10008-1251

**Published:** 2018-08-01

**Authors:** Kirti Singh, Sonal Dangda, Ankush Mutreja, Mainak Bhattacharyya, Kirti Jaisingh

**Affiliations:** 1 Director Professor, Guru Nanak Eye Centre, Maulana Azad Medical College, New Delhi, India; 2 Research Fellow, New York Eye and Ear Infirmary, New York, USA (Formerly Senior Resident, Guru Nanak Eye Centre, Maulana Azad Medical College, New Delhi, India); 3 Consultant, Eye4u Ophthalmic Centre, New Delhi (Formerly Senior Resident, Guru Nanak Eye Centre, Maulana Azad Medical College, New Delhi, India); 4 Senior Resident, Guru Nanak Eye Centre, Maulana Azad Medical College, New Delhi, India; 5 Senior Resident, Guru Nanak Eye Centre, Maulana Azad Medical College, New Delhi, India

**Keywords:** Case report, Developmental glaucoma, KTW syndrome, Naevus of Ota, PPV, Port-wine stain.

## Abstract

**Aim:**

To report a case of bilateral phacomatosis pigmentovascularis (PPV), in a young male,presenting with developmental glaucoma and high myopia along with systemic features of klippel trenanauy weber (KTW) syndrome.

**Background:**

The co-existence of oculodermal melanocytosis (ODM)and port-wine stain was termed PPV by Ota. Port-wine stain presents as part of Sturge-Weber syndrome (SWS). KTW presents with varicose veins and tissue hypertrophy alongwith port-wine stain.

**Case Description:**

A 22-year-old male presented with decreased vision owing to high myopia and advanced glaucoma. Incidental findings noted were pigmentary naevi along with facial port-wine stain, which collectively comprises PPV. Also noted were bilateral varicose veins which are usually seen in association with KTW. In view of advanced visual field damage and inability to control intraocular pressures (IOP) on topical medications, he underwent Glaucoma filtration surgery in both eyes. Intra-operatively care was taken to avoid sudden decompression by controlled anterior chamber paracentesis, and scleral flap closure with releasable sutures was done to prevent hypotony related complications in the immediate postoperative period. Such precautions lead to an uneventful postoperative recovery, and even at 3 years’ follow-up, the patient is maintaining IOP in early teens along with a stable visual acuity and visual fields.

**Conclusion:**

This case highlights the overlapping features of congenital conditions like oculodermal melanocystosis (ODM), SWS, KTW; presenting in a young male. Systemic features reported less frequently with PPV, included palatal pigmentation and palatal vascular malformations.

**Clinical Significance:**

This case re-emphasizes a common origin of these entities, PPV and KTW, from the neural crest cells. Early recognition of the systemic features and timely surgical intervention under appropriate precautions, can be vision salvaging in such cases of developmental glaucoma.

**How to cite this article:** Singh K, Dangda S, Mutreja A, Bhattacharyya M, Jaisingh K. Bilateral Phacomatosis Pigmentovascularis in a Young Male with Developmental Glaucoma and Varicose Veins. J Curr Glaucoma Pract 2018;12(2):94-98.

## BACKGROUND

The co-existence of pigmentary naevi and cutaneous vascular malformations was termed PPV by Ota in 1947.^1^ Described independently, the cutaneous vascular malformations, popularly called as a port-wine stain, are present in the distribution of trigeminal nerve and are usually noted as a part of SWS. The pigmentary naevi or ODM, commonly referred to as naevus of Ota, are dermal melanocytic hamartomas seen as bluish hyperpigmenta-tion along both ophthalmic and maxillary branches of the trigeminal nerve and less commonly mandibular. The condition is common in Asians and females, with bilateral cases being rare.^2^ Glaucoma occurs in half of the patients with SWS and in 10% of those with ODM.^3,4^ KTW, although a separate entity, presents with an overlapping feature of port-wine stain along with varicose veins and tissue hypertrophy.^5,6^

We report a case of bilateral PPV with developmental glaucoma, ONH pigmentation and high myopia in association with varicose veins, a feature seen in KTW. We also aim to highlight that early diagnosis and timely surgical intervention can prevent glaucoma blindness in these patients.

## CASE DESCRIPTION

A young male, 22 years old, presented to our tertiary eye center with complaints of a painles, gradual decrease in vision both eyes, more in the right for 1 year duration. He gave a history of wearing high power glasses (-12.0 DS) OU since early childhood for distance vision.

**Figs. 1 A to E:**
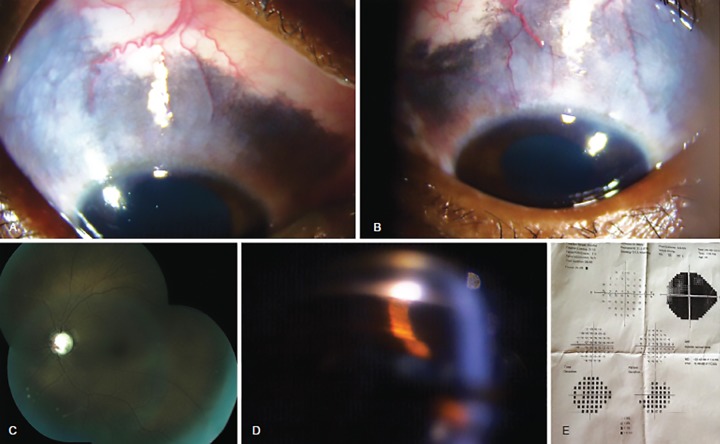
(A) and (B) Slit lamp photograph OD and OS respectively showing scleral hyperpigmentation and prominent episcleral vessels; (C) Fundus montage OS showing 0.8 cupping and pigmentation within infero-temporal margin of optic nerve head. No choroidal or retinal hyperpigmentation seen; (D) Gonioscopy photograph showing wide angle recess with anterior insertion of iris with homogeneously dense trabecular pigmentation and concavity of iris configuration; (E) Visual field 24’2 showing double arcuate pattern with macular split OS

On presentation, best corrected visual acuity (BCVA) was hand movements close to face (HMCF) with inaccurate projection of rays (three quadrants) OD and 6/24 (0.6 logMAR) projection of rays accurate OS. Ocular examination revealed a port-wine stain on the right upper lid along with bluish-black scleral pigmentation and dilated prominent episcleral vessels (episcleral vascular malformations i.e., EVM) in both eyes (Figs 1A and B) with a clear cornea, deep anterior chamber, homogenously dense iris pigmentation and a clear lens. Posterior segment examination revealed both optic nerves to be average in size; OD showed a 0.9 cupping with up to 270^o^ neuro-retinal rim loss; OS showed 0.8 cupping with a bipolar notch. There was pigmentation within the inferotemporal optic disc margin bilaterally, without any associated choroidal or retinal pigmentation (Fig. 1C). The IOP noted was 50 mm Hg OD and 44 mm Hg OS and required systemic hyper-osmotic agents for control. Gonioscopy revealed wide angle recess with an anterior or high insertion of iris reaching up to the anterior trabecular meshwork at places, along with homogeneously dense trabecular pigmentation and concavity of iris configuration (Fig. 1D). The visual fields were not possible in the right eye due to poor vision while advanced field loss, i.e., incomplete double arcuate scotoma with the involvement of fixation was seen in the left eye (Fig. 1E).

**Figs 2 A to C:**
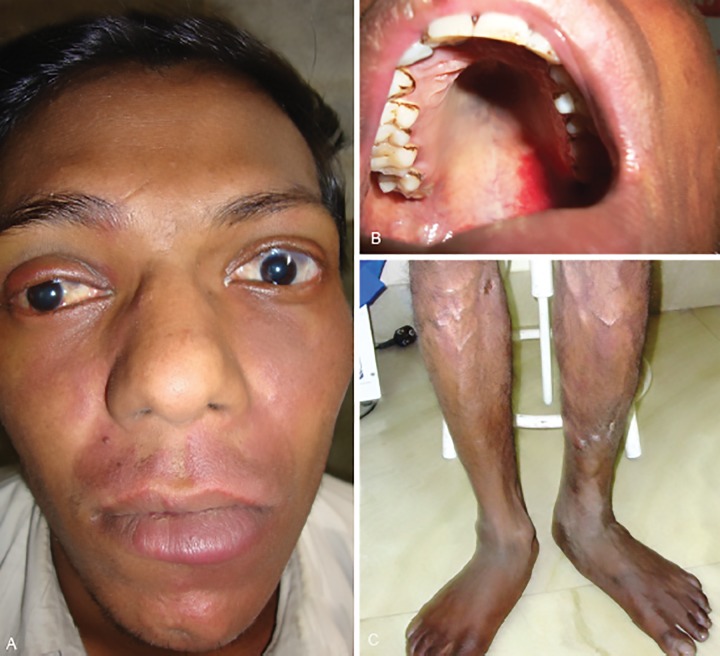
(A) Bilateral port-wine stain on face, over cheek, upper jaw and chin, more on left side alongwith brownish-black pigmentation over temples and forehead extending onto cheek and medial aspect of lower lid bilaterally and also upper jaw on left side; (B) Palatal Melanocytosis centrally along with Palatal vascular malformation on the left side; (C) Bilateral lower limb varicose veins

On systemic examination, bilateral port-wine stain could be noted on the face, over the cheek, upper jaw and chin, more on the left side along with brownish-black pigmentation over temples and forehead extending onto cheek and medial aspect of lower lid bilaterally and also upper jaw on the left side (Fig. 2A). Similar pigmentation was seen on the hard palate (Palatal Melanocytosis i.e., PM) centrally along with palatal vascular malformation (PVM) on the left side (Fig. 2B). No other port-wine nevi were present on any other body parts. The facial and ocular pigmentation (ODM) had been present since early childhood. There was no history of seizures, hemiparesis or mental retardation. Bilateral varicose veins were noted over the last ten years requiring surgical intervention one year prior (Fig. 2C). Neuroimaging by MRI could not document cerebral calcification or atrophy. Family history for similar disposition was negative, thus pointing to a sporadic inheritance.

Due to advanced glaucomatous damage in both eyes and requirement of systemic anti-glaucoma medications for IOP control, the patient was subjected to trabeculec-tomy with anti-fibrotics and releasable sutures in both eyes sequentially at 2 weeks interval. Intraoperatively, care was taken to stabilize the IOP by systemic hyperosmotic agents and controlled paracentesis was done to maintain a stable anterior chamber and help reduce the risk of suprachoroidal hemorrhage. The surgery was uneventful, without the need for draining sclerotomies, but a mild hyphaema occurred at the very end, due to episcleral vessel bleed while suturing the scleral flap. Post-operative course was uneventful, and complications like hypotony, shallow chamber, fresh bleeds or high IOP spikes were not noted. The releasable sutures were removed at two weeks post-operatively. Trabecular meshwork was sent for his-topathology and noted to have increased melanocytes. By 6 weeks’ post-operative, his vision improved to finger counting 1 meter with -14DS OD and 6/18 with -11DS OS; which has remained stable over 3 years follow-up. The IOP is maintained between 9 to 13 mm Hg without any medications.

## DISCUSSION

ODM, mostly seen in Asians, classically presents with hyperpigmentation of the periocular skin, episclera, mucous membranes and facial skin in the distribution of trigeminal nerve (ophthalmic and maxillary divisions). The lesion consists of scattered groups of fusiform cells, within the deeper dermis, containing large melanin granules. Ocular involvement in dermatological series varies from 22 to 76%, with reported 63% in a large ophthalmic series, majorly involving the episclera, iris, trabecular meshwork, and choroid, with 10% manifesting glaucoma.^4^ Port-wine stain in SWS is composed of ectatic blood vessels in the dermis, which does not resolve over time. Ocular involvement with similar lesion leads to the development of glaucoma in almost half of these patients.^3^ While, the presence of leptomeningeal angioma on the ipsilateral surface of cerebral hemisphere is responsible for the development of seizures and mental retardation in SWS patients. PPV is the simultaneous occurrence of ODM and port-wine stain. KTW is although a separate syndrome presenting with varicose veins, is related to PPV by virtue of common origin and presence of port-wine stain.^5,6^

All above are disorders of neural crest cell migration and differentiation involving the vasomotor nerve cells and abnormal melanocytes which originate there.^7,8^ Although amply reported in isolation of each other, a combination of overlapping systemic features along with glaucoma has been reported less, bilateral presentations are fewer still.^9-14^ Single extensive series noted in Thai population by Teekhasaenee and Ritch comprised of nine patients with combined oculodermal hyperpigmentation and vascular malformations with glaucoma, of which six were female, and four patients had combined KTW with PPV presenting as congenital glaucoma at 1-2 months age, of which only one had a bilateral presentation.^7^ The present case differs from the above series by presenting in the third decade in a male patient. Although onset is unclear, it is unlikely to becongenital glaucoma as features like enlarged corneal diameters, corneal scarring or limbal stretching are absent. The worse eye, i.e., right eye shows vascular malformation of the ipsilateral upper lid (Fig. 2A). The association with high myopia of -12.0 D has not been reported previously, and presence of optic nerve head pigmentation is extremely rare.^2^

Glaucoma in patients with PPV is said to be multifactorial in origin.^4,7,12,15^ The various mechanisms could be immature angle structures, increased trabecular pigmentation, the presence of excessive melanocytes in the trabecular meshwork and raised episcleral venous pressure. Immaturity of angle has also been confirmed histopathologically in one patient with KTW.^12^ Such glaucoma is often unresponsive to medical treatment, as was seen in the present case. Even surgical intervention in these cases is fraught with complications like suprachoroidal hemorrhage, choroidal effusions, and hypotony. Preventive posterior sclerotomy has been suggested for the same.^16^ However, its necessity as a prophylactic measure to prevent the occurrence of the said complications in these patients has been questioned with reports of safe glaucoma filtration surgeries even without it.^17^ Mandal et al. noted that patients having choroidal hemangioma are more prone to these complications and it may be advisable to look for the same and decide accordingly instead of attempting a prophylactic posterior sclerotomy in all.^18^ In this case, as well, successful trabeculectomy could be achieved without prophylactic posterior drainage sclerotomies. At the same time, essential precautions to be observed include pre-operative stabilization of IOP with systemic hyper-osmotic agents and oral acetazolamide and slow decompression before attempting sclerostomy. Releasable sutures for scleral flap closure guard against problems related to hypotony in the immediate postoperative period.^19^

Thus, definitive and early surgical intervention, where required, should not be deferred in such patients only because of the surgeons’ fear of intraoperative and postoperative complications. Although these conditions are difficult to manage, awareness about problems likely to be encountered and necessary precautions needed to minimize the same, can help maintain vision in these cases.

## CONCLUSION

This case of bilateral ocular melanocytosis showed the involvement of sclera, trabecular meshwork and optic nerve head along with myopia and developmental glaucoma. Systemically it was associated with bilateral naevus of Ota with port-wine stain, along with palatal pigmentation and palatal vascular malformation and varicosity of veins in lower limbs bilaterally. This case re-emphasizes a common origin of these entities, i.e., PPV and KTW, from the neural crest cells and highlights that they may present simultaneously with overlapping features.

## CLINICAL SIGNIFICANCE

Early recognition of the ocular and systemic manifestations of these disorders is essential and definitive management initiated early in the course of the disease can help prevent blindness. Thus, a timely surgical intervention, under appropriate precautions, can be vision salvaging in such cases of developmental glaucoma.
